# Notification that new names and new combinations have appeared in volume 63, part 6, of the IJSEM

**DOI:** 10.1099/ijs.0.055343-0

**Published:** 2013-09

## Abstract

This listing of names published in a previous issue of the IJSEM is provided as a service to bacteriology to assist in the recognition of new names and new combinations. This procedure was proposed by the Judicial Commission [Minute 11(ii), *Int J Syst Bacteriol* **41** (1991), p. 185]. The names given herein are listed according to the Rules of Priority (i.e. page number and order of valid publication of names in the original articles). Taxonomic opinions included in this list (i.e. the creation of synonyms or the emendation of circumscriptions) cannot be considered as validly published nor, in any other way, approved by the International Committee on Systematics of Prokaryotes and its Judicial Commission.

**Table:** 

Name/author(s):	Proposed as:	Page no.
*Oscillibacter ruminantium* Lee *et al.* 2013	sp. nov.	1944
*Fonticella* Fraj *et al.* 2013	gen. nov.	1949
*Fonticella tunisiensis* Fraj *et al.* 2013	sp. nov.	1949
*Tamlicoccus* Lee 2013	gen. nov.	1953
*Tamlicoccus marinus* Lee 2013	sp. nov.	1953
*Belnapia soli* Jin *et al.* 2013	sp. nov.	1958
*Cloacibacillus* Ganesan *et al.* 2008 emend. Looft *et al.* 2013	emend.*	1964
*Cloacibacillus porcorum* Looft *et al.* 2013	sp. nov.	1964
*Dissulfuribacter* Slobodkin *et al.* 2013	gen. nov.	1970
*Dissulfuribacter thermophilus* Slobodkin *et al.* 2013	sp. nov.	1971
*Polycladomyces* Tsubouchi *et al.* 2013	gen. nov.	1979
*Polycladomyces abyssicola* Tsubouchi *et al.* 2013	sp. nov.	1979
*Roseimicrobium* Otsuka *et al.* 2013	gen. nov.	1984
*Roseimicrobium gellanilyticum* Otsuka *et al.* 2013	sp. nov.	1984
*Brevundimonas abyssalis* Tsubouchi *et al.* 2013	sp. nov.	1991
*Sungkyunkwania* Yoon *et al.* 2013	gen. nov.	1998
*Sungkyunkwania multivorans* Yoon *et al.* 2013	sp. nov.	1999
*Mesorhizobium qingshengii* Zheng *et al.* 2013	sp. nov.	2006
*Snodgrassella* Kwong and Moran 2013	gen. nov.	2016
*Snodgrassella alvi* Kwong and Moran 2013	sp. nov.	2016
*Orbales* Kwong and Moran 2013	ord. nov.	2016
*Orbaceae* Kwong and Moran 2013	fam. nov.	2016
*Gilliamella* Kwong and Moran 2013	gen. nov.	2016
*Gilliamella apicola* Kwong and Moran 2013	sp. nov.	2016
*Arcanobacterium phocisimile* Hijazin *et al.* 2013	sp. nov.	2023
*Litorisediminicola* Yoon *et al.* 2013	gen. nov.	2028
*Litorisediminicola beolgyonensis* Yoon *et al.* 2013	sp. nov.	2029
*Flavobacterium noncentrifugens* Zhu *et al.* 2013	sp. nov.	2035
*Algibacter aquimarinus* Park *et al.* 2013	sp. nov.	2041
*Algibacter pectinivorans* (Yi *et al.* 2011) Park *et al.* 2013 (basonym *Pontirhabdus pectinivorans* Yi *et al.* 2011)†	comb. nov.	2042
*Mangrovimonas* Li *et al.* 2013	gen. nov.	2046
*Mangrovimonas yunxiaonensis* Li *et al.* 2013	sp. nov.	2047
*Caldicoprobacter guelmensis* Bouanane-Darenfed *et al.* 2013	sp. nov.	2051
*Domibacillus* Seiler *et al.* 2013	gen. nov.	2060
*Domibacillus robiginosus* Seiler *et al.* 2013	sp. nov.	2060
*Extensimonas* Zhang *et al.* 2013	gen. nov.	2065
*Extensimonas vulgaris* Zhang *et al.* 2013	sp. nov.	2065
*Halanaerobium sehlinense* Abdeljabbar *et al.* 2013	sp. nov.	2073
*Flavobacterium anatoliense* Kacagan *et al.* 2013	sp. nov.	2078
*Flavobacterium ceti* Vela *et al.* 2007 emend. Kacagan *et al.* 2013	emend.*	2079
*Desulfotomaculum peckii* Jabari *et al.* 2013	sp. nov.	2085
*Mariniradius* Bhumika *et al.* 2013	gen. nov.	2090
*Mariniradius saccharolyticus* Bhumika *et al.* 2013	sp. nov.	2091
*Aquiflexum* Brettar *et al.* 2004 emend. Bhumika *et al.* 2013	emend.*	2092
*Aquiflexum balticum* Brettar *et al.* 2004 emend. Bhumika *et al.* 2013	emend.*	2092
*Sinobacterium* Su *et al.* 2013	gen. nov.	2097
*Sinobacterium caligoides* Su *et al.* 2013	sp. nov.	2098
*Amphritea japonica* Miyazaki *et al.* 2008 emend. Su *et al.* 2013	emend.*	2098
*Sagittula* Gonzalez *et al.* 1997 emend. Lee *et al.* 2013	emend.*	2105
*Sagittula marina* Lee *et al.* 2013	sp. nov.	2105
*Burkholderia grimmiae* Tian *et al.* 2013	sp. nov.	2111
*Parvularcula dongshanensis* Yu *et al.* 2013	sp. nov.	2116
*Massilia lurida* Luo *et al.* 2013	sp. nov.	2120
*Pontimonas* Jang *et al.* 2013	gen. nov.	2127
*Pontimonas salivibrio* Jang *et al.* 2013	sp. nov.	2127
*Falsirhodobacter* Subhash *et al.* 2013	gen. nov.	2134
*Falsirhodobacter halotolerans* Subhash *et al.* 2013	sp. nov.	2136
*Xiangella* Wang *et al.* 2013	gen. nov.	2142
*Xiangella phaseoli* Wang *et al.* 2013	sp. nov.	2142
*Corynebacterium propinquum* Riegel *et al.* 1994 emend. Bernard *et al.* 2013	emend.*	2150
*Palaeococcus pacificus* Zeng *et al.* 2013	sp. nov.	2158
*Novosphingobium lindaniclasticum* Saxena *et al.* 2013	sp. nov.	2165
*Comamonas jiangduensis* Sun *et al.* 2013	sp. nov.	2171
*Acrocarpospora phusangensis* Niemhom *et al.* 2013	sp. nov.	2177
*Salinisphaera* Antunes *et al.* 2003 emend. Shimane *et al.* 2013‡	emend.*	2182
*Salinisphaera japonica* Shimane *et al.* 2013	sp. nov.	2182
*Sphingopyxis indica* Jindal *et al.* 2013	sp. nov.	2190
*Halopelagius fulvigenes* Liu *et al.* 2013	sp. nov.	2195
*Modestobacter* Mevs *et al.* 2000 emend. Qin *et al.* 2013	emend.*	2200
*Modestobacter roseus* Qin *et al.* 2013	sp. nov.	2200
*Acidovorax wautersii* Vaneechoutte *et al.* 2013	sp. nov.	2205
*Glycocaulis* Abraham *et al.* 2013	gen. nov.	2212
*Glycocaulis abyssi* Abraham *et al.* 2013	sp. nov.	2212
*Methylophaga nitratireducenticrescens* Villeneuve *et al.* 2013	sp. nov.	2220
*Methylophaga frappieri* Villeneuve *et al.* 2013	sp. nov.	2221
*Spirochaeta sphaeroplastigenens* Vishnuvardhan Reddy *et al.* 2013	sp. nov.	2227
*Rubrivirga* Park *et al.* 2013	gen. nov.	2232
*Rubrivirga marina* Park *et al.* 2013	sp. nov.	2232
*Thalassolituus marinus* Choi and Cho 2013	sp. nov.	2237
*Flavobacterium haoranii* Zhang *et al.* 2010 emend. Sheu *et al.* 2013	emend.*	2244
*Flavobacterium cauense* Qu *et al.* 2009 emend. Sheu *et al.* 2013	emend.*	2244
*Flavobacterium terrae* Weon *et al.* 2007 emend. Sheu *et al.* 2013	emend.*	2244
*Flavobacterium aquatile* (Frankland and Frankland 1889) Bergey *et al.* 1923 (Approved Lists 1980) emend. Sheu *et al.* 2013	emend.*	2244
*Flavobacterium squillarum* Sheu *et al.* 2013	sp. nov.	2244
*Marinicauda* Zhang *et al.* 2013	gen. nov.	2251
*Marinicauda pacifica* Zhang *et al.* 2013	sp. nov.	2251
*Geodermatophilus telluris* Montero-Calasanz *et al.* 2013	sp. nov.	2258
*Sphingobacterium caeni* Sun *et al.* 2013	sp. nov.	2263
*Paramoritella* Hosoya *et al.* 2009 emend. Yang *et al.* 2013	emend.*	2267
*Paramoritella alkaliphila* Hosoya *et al.* 2009 emend. Yang *et al.* 2013	emend.*	2267
*Paramoritella sediminis* Yang *et al.* 2013	sp. nov.	2267
*Aeromonas australiensis* Aravena-Román *et al.* 2013	sp. nov.	2274
*Hoeflea suaedae* Chung *et al.* 2013	sp. nov.	2279
*Methylomonas paludis* Danilova *et al.* 2013	sp. nov.	2286
*Desulfotomaculum defluvii* Krishnamurthi *et al.* 2013	sp. nov.	2293
*Undibacterium terreum* Liu *et al.* 2013	sp. nov.	2299
*Mycobacterium parakoreense* Kim *et al.* 2013	sp. nov.	2305
*Lonsdalea quercina* subsp*.* *populi* Tóth *et al.* 2013	subsp. nov.	2312
*Blastopirellula cremea* Lee *et al.* 2013	sp. nov.	2318
*Muricauda zhangzhouensis* Yang *et al.* 2013	sp. nov.	2322
*Roseomonas aerophila* Kim *et al.* 2013	sp. nov.	2336
*Vogesella* Grimes *et al.* 1997 emend. Subhash *et al.* 2013	emend.*	2342
*Vogesella alkaliphila* Subhash *et al.* 2013	sp. nov.	2342
*Glaciimonas singularis* Chung *et al.* 2013	sp. nov.	2348

* Taxonomic opinion.

† Park *et al.* (2013) propose to transfer *Pontirhabdus pectinivorans* Yi *et al.* 2011 (the type species of the genus *Pontirhabdus*) to the genus *Algibacter* as *Algibacter pectinivorans* (Yi *et al.* 2011) Park *et al.* 2013, comb. nov. According to Rule 37a, bacteriologists adhering to this proposal must change the name *Pontirhabdus* to *Algibacter*.

‡ In the paper by Shimane *et al.* (2013), ‘Emended description of the genus *Salinisphaera* Antunes *et al*. 2003’ is erroneously cited as ‘Emended description of the genus *Salinisphaera* Crespo-Medina *et al*. 2009’.

